# The Lipopeptide MALP-2 Promotes Collateral Growth

**DOI:** 10.3390/cells9040997

**Published:** 2020-04-16

**Authors:** Kerstin Troidl, Christian Schubert, Ann-Kathrin Vlacil, Ramesh Chennupati, Sören Koch, Jutta Schütt, Raghav Oberoi, Wolfgang Schaper, Thomas Schmitz-Rixen, Bernhard Schieffer, Karsten Grote

**Affiliations:** 1 Max-Planck-Institute for Heart and Lung Research, 61231 Bad Nauheim, Germany; ramesh.chennupati@mpi-bn.mpg.de (R.C.); wolfgang.schaper@mpi-bn.mpg.de (W.S.); 2 Department of Vascular and Endovascular Surgery, University Hospital Frankfurt, 60488 Frankfurt, Germany; christian.schubert@mpi-bn.mpg.de (C.S.); schmitz-rixen@em.uni-frankfurt.de (T.S.-R.); 3 Cardiology and Angiology, Philipps-University Marburg, 35043 Marburg, Germany; ann-kathrin.koch@staff.uni-marburg.de (A.-K.V.); Kochsoe@students.uni-marburg.de (S.K.); j.lamle@gmx.de (J.S.); oberoi.raghav@gmail.com (R.O.); bernhard.schieffer@staff.uni-marburg.de (B.S.); grotek@staff.uni-marburg.de (K.G.)

**Keywords:** TLR2/6, femoral artery ligation, blood flow recovery, collateral growth

## Abstract

Beyond their role in pathogen recognition and the initiation of immune defense, Toll-like receptors (TLRs) are known to be involved in various vascular processes in health and disease. We investigated the potential of the lipopeptide and TLR2/6 ligand macrophage activating protein of 2-kDA (MALP-2) to promote blood flow recovery in mice. Hypercholesterolemic apolipoprotein E (Apoe)-deficient mice were subjected to microsurgical ligation of the femoral artery. MALP-2 significantly improved blood flow recovery at early time points (three and seven days), as assessed by repeated laser speckle imaging, and increased the growth of pre-existing collateral arteries in the upper hind limb, along with intimal endothelial cell proliferation in the collateral wall and pericollateral macrophage accumulation. In addition, MALP-2 increased capillary density in the lower hind limb. MALP-2 enhanced endothelial nitric oxide synthase (eNOS) phosphorylation and nitric oxide (NO) release from endothelial cells and improved the experimental vasorelaxation of mesenteric arteries ex vivo. In vitro, MALP-2 led to the up-regulated expression of major endothelial adhesion molecules as well as their leukocyte integrin receptors and consequently enhanced the endothelial adhesion of leukocytes. Using the experimental approach of femoral artery ligation (FAL), we achieved promising results with MALP-2 to promote peripheral blood flow recovery by collateral artery growth.

## 1. Introduction

Cardiovascular diseases are still one of the most common causes of morbidity and mortality worldwide. In this regard, atherosclerosis—a chronic inflammatory disease of the arteries—has long been identified as the underlying cause that could ultimately lead to fatal events such as myocardial infarction, strokes [1] and also to peripheral artery disease (PAD) [2]. Atherosclerosis is characterized as a progressing process of plaque growth in the arterial vessel wall that develops in the setting of hyperlipidemia and goes along with vascular lumen stenosis, plaque rupture and erosion [3]. The growth of pre-existing collateral arteries (also termed as arteriogenesis) represents an endogenous mechanism of bypassing occluded vessels and is an important adaptive response to maintain or restore arterial perfusion [4]. Arteriogenesis occurs in tissues near to arterial stenosis whereas down-stream ischemic regions undergo angiogenesis, which is the growth of new capillaries. Collateral growth is driven by hemodynamic forces such as shear stress [5,6] and wall stress and leads to initial vasodilation due to increased levels of nitric oxide [7]. It is the reason why significant stenoses of main arteries may remain asymptomatic in patients for some time. However, in most cases, collateral growth could not ensure sufficient blood supply to the affected region, which becomes ischemic over time. Therefore, developing therapeutic approaches to improve this process is certainly desirable.

Just like atherosclerosis, collateral growth is critically driven by inflammatory processes. Chemokines, such as CC-chemokine ligands (CCL)2, and adhesion molecules, such as intercellular adhesion molecules (ICAM)-1, mediate the recruitment and accumulation of mainly monocytes into the arterial wall at sites of collateral growth. The proliferation of endothelial cells and smooth muscle cells subsequently lead to the lumen size expansion of the affected collateral artery [8]. In recent years, we have successfully used the Toll-like receptor (TLRs) 2/6 agonist macrophage activating protein of 2-kDA (MALP-2) to boost inflammatory processes and promote adaptive and regenerative mechanisms. TLRs belong to the class of pattern recognition receptors which were initially discovered on mammalian immune cells and recognize conserved pathogen-associated molecular patterns in order to initiate the immune response and combat bacterial infections [9]. In addition, an important role of TLRs has emerged later in many physiological as well as pathophysiological processes. For example, during atherogenesis, pattern recognition receptors such as TLRs are involved in the induction of inflammatory processes in response to exogenous and endogenous ligands which arise after necrotic cell death or extracellular matrix degradation [10]. MALP-2 is a common diacylated bacterial lipopeptide which is recognized by a heterodimer of TLR2 and TLR6 and was originally described as a potent activator of macrophages [11,12,13]. We recently reported that a single application of MALP-2 triggers beneficial vascular effects such as angiogenesis [14], endothelial wound healing and the inhibition of neointima formation following vascular injury [15]. Additionally, we observed the augmented angiogenic potential of mesenchymal stem cells after MALP-2 treatment in a sheep model of tissue engineering [16]. Given the importance of inflammatory processes for collateral growth—and because we had already established vascular cells as suitable target cells for MALP-2—we next investigated the potential of MALP-2 to promote blood flow recovery after the experimental ligation of the femoral artery by collateral growth in mice.

## 2. Materials and Methods

### 2.1. Reagents and Antibodies

The macrophage-activating lipopeptide of 2 kDa (MALP-2) was synthesized and purified as described before [11]. Fibronectin was purchased from Promocell (Heidelberg, Germany), calcein-AM from eBioscience (San Diego, CA, USA), 4′,6-diamidino-2-phenylindole (DAPI) from Sigma-Aldrich (Munich, Germany). Phenylephrine (PE), acetylcholine (ACh), noradrenaline and N-Nitroarginine methyl ester (L-NAME) were purchased from Sigma-Aldrich. Indomethacin was obtained from Alfa Aaesar (Thermo Fisher Scientific, Waltham, MA, USA), sodium nitroprusside from Honeywell (Seelze, Germany) and U46619 from Cayman Chemical (Ann Arbor, MI, USA). Antibodies for immunofluorescence against CD68, CD31 and Ki67 were from Abcam (Cambridge, UK) and against α-SMA-Cy3 were from Sigma-Aldrich. Antibodies for Western blot against VCAM-1 and β-Actin were from Santa Cruz (Dallas, TX, USA) and against p-AKT (S473), AKT, p-eNOS (S1177) and eNOS were from Cell Signaling Technology (Danvers, MA, USA). Appropriate secondary antibodies for immunofluorescence and Western blot were purchased from Thermo Fisher Scientific (Waltham, MA, USA).

### 2.2. Mice and Cells

The animal handling and all experimental procedures were in accordance with the guidelines from Directive 2010/63/EU of the European Parliament on the protection of animals used for scientific purposes and were approved by the Animal Care and Use Committee of the state Hessen (approval reference numbers V54-19c20/15-B2/1152 (23.05.17); B2-1077 (29.07.16)). For femoral artery ligation (FAL), 8–12-week-old male C57BL/6 and BALB/c mice were purchased from Charles River (Sulzfeld, Germany). Six to ten-week-old male Apoe-deficient mice with a C57BL/6 background from our own breeding were fed a high fat diet (HFD, 21% butterfat, 1.5% cholesterol, Ssniff, Soest, Germany) for 12 weeks and operated on thereafter. Adductor muscles were isolated from the left and right upper hind limbs of 10-week-old male C57BL/6 mice, cut into 1–2 mm pieces with fine scissors and 4 pieces were placed in a well of a 96-well plate for ex vivo stimulation with MALP-2. The endothelial MyEnd cell line was grown in Dulbecco’s modified Eagle medium (DMEM, Gibco, Darmstadt, Germany) with 10% fetal calf serum (FCS, PAN-Biotech, Aidenbach, Germany) and 1% penicillin/streptomycin (100 U/mL and 100 mg/mL, Sigma-Aldrich). The MyEnd cells showed typical endothelial properties and, as they grew to complete confluence, were highly positive for the endothelial marker CD31 and expressed the MALP-2 receptors TLR2 and TLR6 (Figure S1). The monocyte/macrophage cell line J774A.1 was grown in DMEM-Glutamax (Gibco) with 10% FCS and 1% penicillin/streptomycin (P/S).

### 2.3. Experimental Femoral Artery Ligation (FAL)

The mice were subjected to FAL as described elsewhere [17]. During the surgical procedure, the mice were under general anesthesia with isoflurane (2.5% for induction, 1.5–2.0% maintenance). After the FAL, the mice were intravenously injected with MALP-2 (1 µg in 125 µL phosphate-buffered saline (PBS) per mouse) or vehicle control (125 µL PBS). For postoperative analgesia, carprofen (5 mg/kg body weight) was subcutaneously injected once prior to surgery. The contralateral leg served as the control. After the termination of experiments, the mice were euthanized by an anesthetic overdose.

### 2.4. Laser Speckle Imaging

The perfusion of the hind paws was assessed using a laser speckle imaging device (moorFLPI-2; software for acquisition and MoorFLPI Review V5.0 for evaluation, Moor Instruments, Axminster, UK) on a heating plate (37 °C) before the FAL (d0 pre), immediately after (d0 post), and d3, d7 and d10 after the FAL.

### 2.5. Histology and Immunohistochemistry

The mice were perfused with 10 mL of a vasodilation buffer (100 µg adenosine, 1 µg sodium nitroprusside, 0.05% bovine serum albumin in PBS, pH 7.4), followed by 10 mL of 3% paraformaldehyde post mortem. Tissue from the ligated left and the not ligated right adductor muscles was harvested and placed in 15% sucrose in PBS for 4 h and overnight at 4 °C in 30% sucrose in PBS. The tissue was cryopreserved in Tissuetek (Sakura Finetek, Staufen, Germany) and cut into 8 µm cryosections. A morphometric analysis was performed using haematoxilin-eosin staining to evaluate the dimensions of the collateral arteries with the help of ImageJ software (National Institutes of Health, Bethesda, MD, USA). The cryosections were fixed with 5% paraformaldehyde and stained with antibodies against Ki-67, CD31, α-SMA or CD68. The slides were covered with Mowiol (Sigma-Aldrich) and analyzed with a confocal microscope (Leica SP5, Leica, Wetzlar, Germany).

### 2.6. Organ Chamber Experiments (Wire Myography)

The male C57BL/6 mice of 10–12 weeks were killed by CO_2_/O_2_ inhalation. The mesenteric artery was dissected free from surrounding fat and connective tissue and directly mounted in a wire myograph (Danish Myo Technology, Aarhus, Denmark) containing Krebs solution (119 mM NaCl, 4.7 mM KCl, 2.5 mM CaCl_2_·2H_2_O, 1.17 mM MgSO_4_·7H_2_O, 20 mM NaHCO_3_, 1.18 mM KH_2_PO_4_, 0.027 mM EDTA, 11 mM glucose). Mesenteric arterial segments (2 mm) were distended to the diameter at which maximal contractile responses to 10 µM noradrenaline could be obtained. The maximal relaxing response to acetylcholine (ACh, 10 µM) was recorded during a contraction induced by 10 µM noradrenaline; arterial segments which showed less than 85% relaxation were discarded from the experiments.

### 2.7. Real-Time PCR

For the analysis of the mRNA expression, the total RNA was isolated using RNA-Solv® Reagent (Omega Bio-tek, Norcross, GA, USA) following the manufacturer’s instructions and reverse-transcribed with SuperScript reverse transcriptase, oligo(dT) primers (Thermo Fisher Scientific), and deoxynucleoside triphosphates (Promega, Mannheim, Germany). Real-time PCR was performed in duplicates in a total volume of 20 µL using Power SYBR green PCR Master Mix (Thermo Fisher Scientific) on a Step One Plus Real-Time PCR System (Applied Biosystems, Foster City, CA, USA) in 96-well PCR plates (Applied Biosystems). The SYBR Green fluorescence emissions were monitored after each cycle. For normalization, the expression of glyceraldehyde 3-phosphate dehydrogenase as housekeeper was determined in duplicates. The gene expression was calculated using the 2^− ΔΔCt^ method. The PCR primers were obtained from Microsynth AG (Balgach, Switzerland) and are available upon request.

### 2.8. Enzyme-Linked Immunosorbent Assay (ELISA)

The supernatant from cultured tissue pieces of the adductor muscles of C57BL/6 mice was analyzed for CCL2, GM-CSF, IL-1α and TNF-α using a mouse-specific ELISA from R&D Systems (Minneapolis, MN, USA) according to the manufacturer’s protocol with the help of an Infinite M200 PRO plate reader (TECAN Instruments, Maennedorf, Switzerland).

### 2.9. Western Blot

The total protein was extracted with a buffer that contained 150 mM NaCl, 1% Triton X-100, 0.5% sodiumdeoxycholate, 0.1% SDS and 50 mM Tris that was supplemented with a protease inhibitor cocktail (Roche, Penzberg, Germany). The total protein content was measured using a protein quantitation assay (Thermo Fisher Scientific) according to the manufacturer’s protocol. The total protein (20 µg) was loaded onto 10% denaturing SDS gel and transferred to 0.45 mm polyvinylidene fluoride membranes (GE Healthcare, Little Chalfont, UK) for immunoblotting. The membranes were blocked with 5% nonfat dry milk (Sigma-Aldrich) and probed with primary antibodies against VCAM-1, β-Actin, p-AKT, AKT, p-eNOS and eNOS, followed by horseradish peroxidase–labeled secondary antibodies. Proteins were detected using a chemiluminescence substrate (Bio-Rad Laboratories, Hercules, USA). The results were documented on a Chemo-star imaging system (INTAS, Göttingen, Germany). The signal intensity of the chemiluminescence was quantified using Quantity One software (Bio-Rad).

### 2.10. Griess Assay

The MyEnd cells were plated in fibronectin-coated wells of a 96-well plate (TPP, Trasadingen, Switzerland) in DMEM with 10% FCS and 1% P/S and grown to complete confluence. The cells were starved in DMEM with 1% FCS and 1% P/S for 16 h and stimulated with MALP-2 (1 µg/mL) for 2 h. The NO levels in each well were measured using a Griess reagent (Sigma-Aldrich) according to the manufacturer’s instructions.

### 2.11. Adhesion Assay

The MyEnd cells were plated in fibronectin-coated wells of a 48-well plate (TPP) in DMEM with 10% FCS and 1% P/S and grown to complete confluence. The cells were starved in DMEM with 1% FCS and 1% P/S for 16 h and stimulated with MALP-2 (1 µg/mL) for 6 h. In parallel, J774A.1 cells were labeled with 5 μM of calcein-AM (Invitrogen, Carlsbad, CA, USA). according to the manufacturer’s instructions. After stimulation, the MyEnd cells were washed twice with 500 μL of PBS per well; 0.5×10^6^ labeled J774A.1 cells in 500 µL of DMEM with 1% FCS were added per well and co-cultured for 1 h in 5% CO_2_ at 37 °C. After co-incubation, each well was washed three times with 500 μL of PBS and 10 high powerfield (HPF) digital images were taken using an Axio Vert.A1 microscope equipped with an AxioCam MRm camera (Carl Zeiss, Microimaging, Jena, Germany). The adhered calcein-AM-labeled J774A.1 cells per HPF image were counted using ImageJ software.

### 2.12. Statistical Analysis

All the data are represented as means ± SEM. The data were compared using the 2-tailed Student t-test for independent samples or by a 1-way ANOVA followed by the Tukey multiple comparison test (GraphPad Prism, version 6.05; GraphPad Software, La Jolla, CA, USA). A value of *P* < 0.05 was considered statistically significant. The numbers of independent experiments are indicated in each figure legend. The real-time PCR was performed in technical duplicates.

## 3. Results

### 3.1. MALP-2 Improved Perfusion Recovery and Collateral Growth in the Hind Limb Following FAL in Hypercholesterolemic Apoe-Deficient Mice

**Figure 1:**
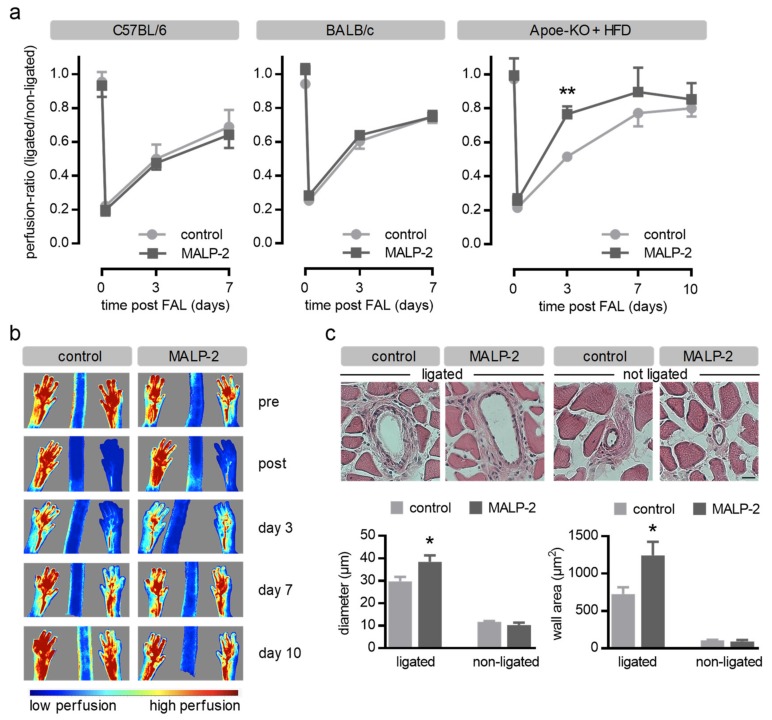
MALP-2 improved the perfusion recovery and collateral growth in the hind limb following femoral artery ligation (FAL) in hypercholesterolemic Apoe-deficient mice. (**a**) Following the FAL, the perfusion recovery was determined by laser Speckle perfusion imaging for C57BL/6, BALB/c and hypercholesterolemic Apoe-KO mice (12 weeks on a high fat diet (HFD)) treated with MALP-2 or PBS (control) pre/post the FAL, after three and seven days and, in Apoe-KO mice, after 10 days. Data are expressed as the ratio of the ligated and the non-ligated hind limb. ** *P* < 0.01, *N* = 4–7. (**b**) Representative laser speckle perfusion images indicate the effect of MALP-2 compared to the control (PBS) on perfusion recovery in the ligated hind limbs of Apoe-KO mice pre/post the FAL and after 3, 7 and 10 days. (**c**) Representative haematoxilin-eosin staining of cross sections of collateral arteries in the adductor muscle of the ligated and the non-ligated hind limbs of hypercholesterolemic Apoe-KO mice treated with MALP-2 or PBS (control) 10 days after the FAL and the corresponding morphometric analysis of the collateral diameter and wall area. Scale bar = 10 µm. * *P* < 0.05 vs. control, *N* = 6–14 collaterals.

Based on our previous findings [14,15,16], we hypothesized that MALP-2 is capable of promoting collateral growth. To analyze the functional effects of systemic MALP-2 application in this regard, the mouse FAL model was applied sequentially to two different wild-type mice strains (C57BL/6 and BALB/c) and additionally to Apoe-deficient mice (Apoe-KO) on a high fat diet (HFD) for 12 weeks. Laser Speckle perfusion measurements were performed prior to and after surgery as well as on days 3 and 7 and, for Apoe-KO mice, on day 10. Following the left FAL, the ratio of left hind limb perfusion compared to that of the hind paw of the non-ligated right site dropped to less than 25% in all groups (Figure 1a). The perfusion recoveries of C57BL/6 and BALB/c wild-type mice which received MALP-2 or PBS (control) were found to be similar on day three and day seven post FAL (Figure 1a). However, MALP-2 significantly improved the perfusion recovery of hypercholesterolemic Apoe-KO mice on day three post FAL. The beneficial effect of MALP-2 on perfusion recovery was limited to early time points and returned to control conditions on day 10 post FAL (Figure 1a,b). Since the functional improvement of MALP-2 in the FAL model was limited to Apoe-KO mice on a HFD, we concluded that hypercholesterolemic conditions with compromised vascular functions are required for the observed beneficial effects of MALP-2; we therefore focused on this model in the following analysis.

The remodeling of the collateral arteries was verified by morphometry in cross sections of the left adductors 10 days after the FAL. The MALP-2 application significantly increased the collateral inner diameter as well as the collateral wall area, thus documenting enhanced collateral growth with MALP-2 (Figure 1c). Since collateral growth is critically influenced by hemodynamic forces, we analyzed the atherosclerotic arterial plaque load in the experimental Apoe-KO mice after 12 weeks of the HFD diet. As expected, we detected plaques in the aortic root and in the thoracoabdominal aorta. However, the atherosclerotic plaque load was not different between the control and the MALP-2-treated group (Figure S2a,b). Plaques in the femoral artery were only detected in rare cases. In addition, we investigated the collateral arteries, which were found to be highly positive for Oil Red O, indicating lipid deposition in the collateral vascular wall in hypercholesterolemic Apoe-deficient mice (Figure S2c). As expected, this was not the case in parallel-performed control Oil Red O staining in collaterals from C57BL/6 mice (Figure S2c). However, atherosclerotic plaques were not detected, excluding the possibility that plaque morphology itself might influence the hemodynamics and thereby collateral remodeling and growth.

### 3.2. MALP-2 Increased Pericollateral Macrophage Accumulation, Endothelial Cell Proliferation and Downstream Angiogenesis Following FAL

**Figure 2:**
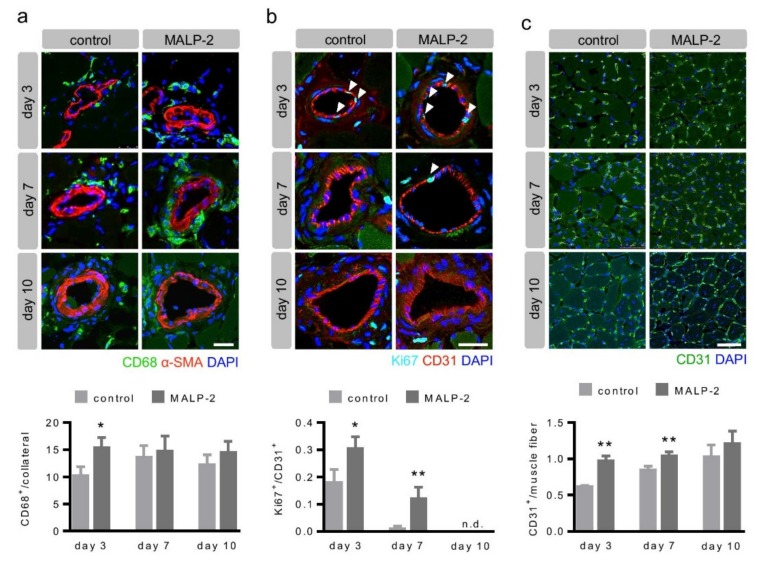
MALP-2 increased pericollateral macrophage accumulation, endothelial cell proliferation and downstream angiogenesis following FAL. This shows the representative immunostaining of cross sections of collateral arteries in the adductor muscle and the calf muscle of the ligated hind limb in hypercholesterolemic Apoe-KO mice treated with MALP-2 and PBS (control) 3, 7 and 10 days after the FAL and the corresponding quantitative analysis. (**a**) CD68 staining to assess the accumulation of macrophages around the collateral (α-SMA indicates the media of the collateral wall). Scale bar = 25 µm. (**b**) Ki67 staining to determine the portion of proliferating CD31-positive collateral endothelial cells (white arrow heads). Scale bar = 25 µm. (**c**) CD31 indicates capillary density in the calf muscle. Scale bar = 50 µm. * *P* <0.05, ** *P* <0.01 vs. control, *N* = up to 20 collaterals, n.d. = not detected.

In order to investigate the influence of MALP-2 on the vascular remodeling process, tissue from the adductor muscles was harvested from hypercholesterolemic Apoe-deficient mice 3, 7 and 10 days following the FAL. In the initial phase, the collateral growth is critically driven by pericollateral macrophage assembly and endothelial proliferation [4,6,18]. MALP-2 significantly increased the macrophage accumulation around the collateral artery on day three after the FAL compared to the control. There was no effect of MALP-2 at later time points (Figure 2a). Likewise, we detected significantly more proliferating endothelial cells in MALP-2-treated mice on days three and seven after the FAL. In general, no proliferating endothelial cells were detected on day 10 (Figure 2b). The stenosis or occlusion of a major arterial conductance vessel entails a reduced blood supply and subsequent ischemia in the downstream supply area. Angiogenesis with an increased capillary density is usually the counteracting adaptive process in tissue ischemia. Therefore, we investigated angiogenesis in the gastrocnemius muscle and found that MALP-2 increased the capillary density on day three and day seven. This was not different anymore on day 10 post FAL (Figure 2c). Our results indicated that the effects of MALP-2 on collateral growth occurred within the first seven days after FAL.

**Figure 3:**
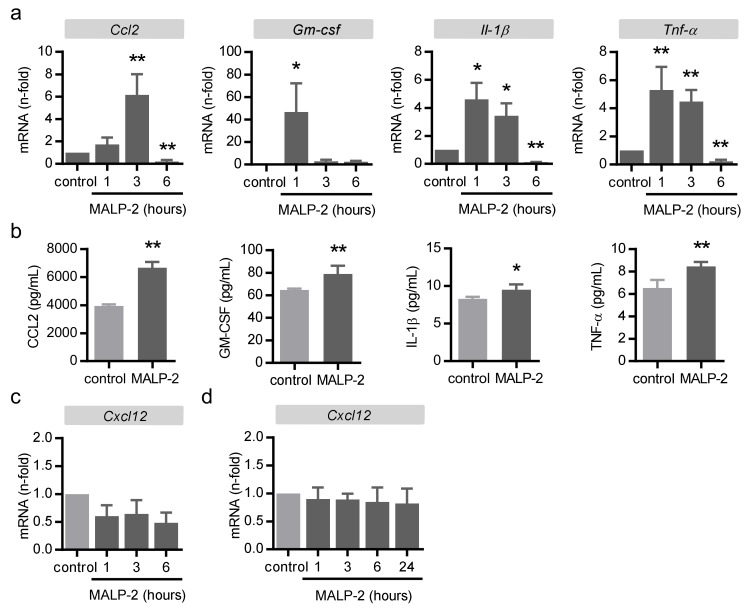
MALP-2 up-regulated inflammatory genes in the upper hind limb muscle. Tissue pieces of the adductor muscles of C57BL/6 mice were isolated and stimulated ex vivo with MALP-2 (1 µg/mL); *Ccl2*, *Gm-csf*, *Il-1β* and *Tnf-α* mRNA levels were analyzed after the indicated times by (**a**) real-time PCR and (**b**) the corresponding protein in the supernatant after 6 h by ELISA. *CXCL12* mRNA levels were analyzed (**c**) in tissue pieces of the adductor muscle of C57BL/6 mice ex vivo and in (**d**) MyEND cells following MALP-2 stimulation (1 µg/mL) after the indicated times by real-time PCR. * *P* < 0.05, ** *P* < 0.01 vs. control, *N* = 4–6.

To explore which factors were potentially involved in the process of MALP-2-induced collateral growth, we stimulated tissue pieces of the adductor muscles from C57BL/6 mice ex vivo with MALP-2. A real-time PCR analysis revealed increased expression levels for the established mediators of collateral growth such as CC-chemokine ligand 2 (*Ccl2*) [19] and granulocyte macrophage colony-stimulating factor (*Gm-csf*) [20] as well as, for the general inflammatory markers, interleukin 1β (*Il-1β*) and tumor necrosis factor-α (*Tnf-α*, Figure 3a). Likewise, the corresponding protein in the supernatant was found to be enhanced (Figure 3b). Das et al. recently reported that the axis of C-X-C motif chemokine ligand 2 (CXCL12, also known as stromal cell-derived factor 1) and its receptor C-X-C motif receptor 4 (CXCR4) is relevant for the injury-induced cardiac collateral growth in neonatal mice and could also be induced by exogenous CXCL12 in adult mice [21]. However, MALP-2 did not induce *Cxcl12* expression ex vivo in the adductor muscle tissue (Figure 3c) or in the cultured MyEnd endothelial cells (Figure 3d), suggesting that this process did not play a role in MALP-2-dependent collateral growth.

### 3.3. MALP-2 Improved NO-Dependent Vascular Relaxation and Enhanced Endothelial Cell-Derived NO Release

**Figure 4:**
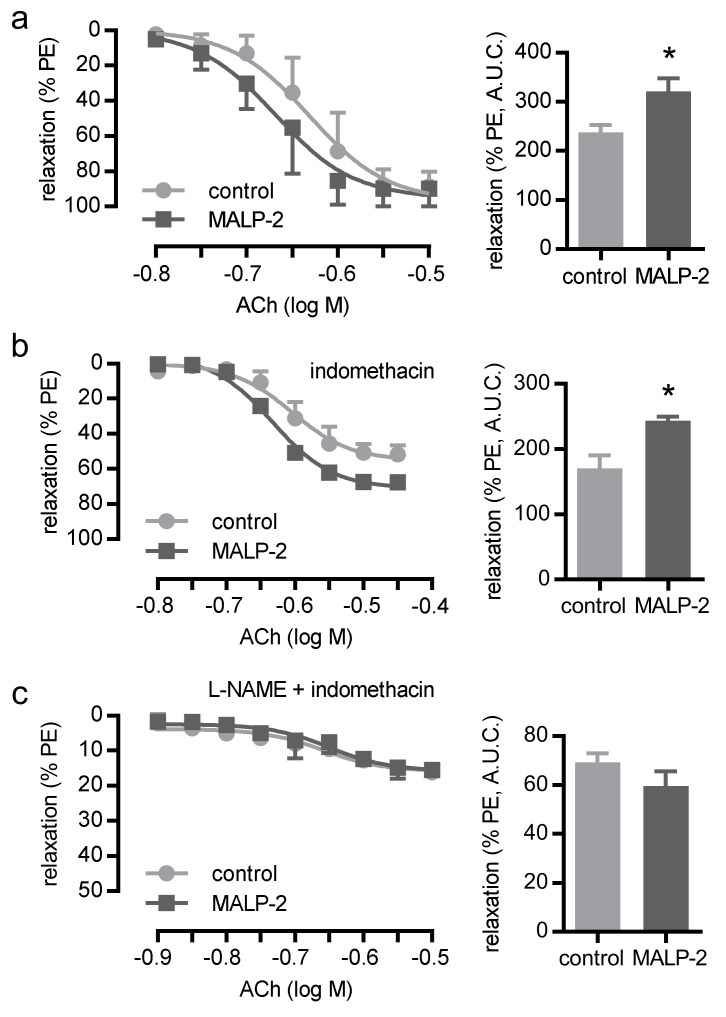
MALP-2 improved NO-dependent vascular relaxation in the mesenteric arteries of C57BL/6 mice. (**a**) The relaxation response to acetylcholine (ACh 0.001–10 µM) during phenylephrine-induced (PE, 10 µM) contraction in mesenteric arteries incubated with MALP-2 or PBS (control), *N* = 6. (**b**) The relaxation response to ACh (0.01–10 µM) during K^+^-induced (60 mM) contraction in mesenteric arteries incubated with indomethacin (10 µM, COX-inhibitor) and MALP-2 or PBS, *N* = 3. (**c**) The relaxation response to ACh (0.001–10 µM) in the presence of L-NAME (100 µM, NOS inhibitor) and indomethacin (10 µM). A.U.C. = area under the curve, * *P* < 0.05 vs. control, *N* = 3.

**Figure 5:**
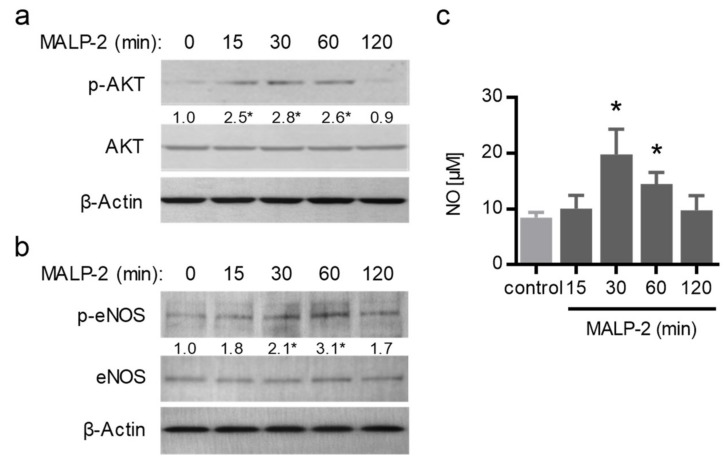
MALP-2 enhanced the endothelial cell-derived NO release. MyEnd cells were stimulated with MALP-2 (1 µg/mL); (**a**) the AKT phosphorylation (p-AKT) as well as (**b**) the eNOS phosphorylation (p-eNOS) were analyzed after the indicated times by Western blot and (**c**) the NO release was analyzed with the Griess reagent. The numbers between panels indicate fold-change vs. unstimulated after normalization to total AKT or eNOS, respectively. β-Actin was used as the loading control. * *P* < 0.05 vs. control, *N* = 4–5.

Since endothelial dysfunction may limit collateral growth itself or the beneficial effects of collateral vessels on tissue perfusion, we assessed the effect of MALP-2 on vascular relaxation. To this end, we isolated mesenteric arteries from C57BL/6 mice to perform wire myography. MALP-2 significantly improved acetylcholine (ACh)-induced relaxation of phenylephrine (PE)-preconstricted (10 µM) mesenteric arteries (Figure 4a). To test for differences in endothelium-derived NO release, we inhibited endothelium-dependent hyperpolarization by depolarizing the vessels with high potassium buffers (60 mM K^+^) and by inhibiting cyclooxygenases using indomethacin. Under these conditions, the relaxing responses to ACh could be entirely attributed to NO [22]. The MALP-2 treatment resulted in significantly increased endothelium-derived NO responses (Figure 4b). This effect completely disappeared when the endothelial nitric oxide synthase (eNOS) was additionally blocked with L-NAME (Figure 4c). Furthermore, MALP-2 also significantly improved the relaxation response in thoracic aorta (data not shown). These results demonstrated a crucial role for endothelium-derived NO in MALP-2-dependent vascular relaxation. Moreover, in MyEnd cells, MALP-2 led to a fast and transient increase in the protein kinase B (also known as AKT) phosphorylation (Figure 5a) and eNOS phosphorylation (Figure 5b) and consequently to an increased NO release (Figure 5c).

### 3.4. MALP-2 Up-Regulated Endothelial Adhesion Molecules and Enhanced the Endothelial Adhesion of Monocytic Cells

**Figure 6:**
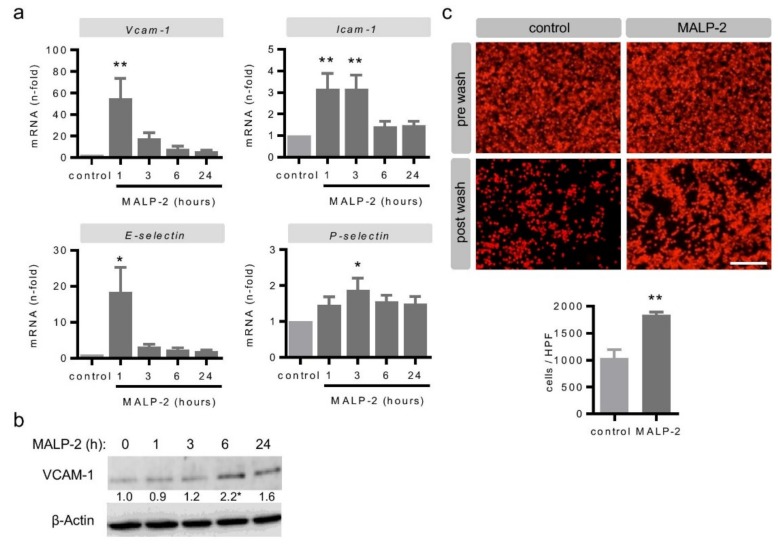
MALP-2 up-regulated endothelial adhesion molecules and enhanced the endothelial adhesion of monocytic cells. (**a**) The MyEnd cells were stimulated with MALP-2 (1 µg/mL) and the VCAM-1, ICAM-1, E-selectin and P-selectin mRNA levels were analyzed after the indicated times by real-time PCR. * *P* < 0.05, ** *P* < 0.01 vs. control, *N* = 6–8. (**b**) The MyEnd cells were stimulated with MALP-2 (1 µg/mL) and the VCAM-1 protein expression was analyzed after the indicated times by Western blot. β-Actin was used as the loading control. The numbers between panels indicate fold-change vs. unstimulated after normalization to β-Actin. * *P* < 0.05 vs. control, *N* = 4–5. (**c**) Fluorescence images depicting calcein-AM-labeled J774A.1 cells on a MyEnd monolayer with or without pretreatment with MALP-2 (1 µg/mL) for 6 h with an additional adhesion time of 1 h and the corresponding quantitative analysis. Pictures before and after washing are shown. Scale bar = 100 µm, ** *P* < 0.01 vs. control, *N* = 3.

Arteriogenesis is a multi-faceted, highly coordinated process involving the endothelial adhesion of monocytes onto endothelial cells [4,6]. To explore the potential underlying mechanism responsible for the positive effects of MALP-2 on collateral growth after FAL, we conducted a series of in vitro experiments. In endothelial MyEnd cells, MALP-2 led to a strong transient increase in the mRNA levels of vascular cell adhesion molecule-1 (*Vcam-1*) after only 1 h (Figure 6a) and slightly delayed to an increase in VCAM-1 protein levels (Figure 6b). The mRNA levels of the other major endothelial adhesion molecules, i.e., intercellular adhesion molecule-1 (*Icam-1*), *E-selectin* and *P-selectin*, were also increased between 1 and 3 h following the MALP-2 stimulation (Figure 6a). In addition, we investigated the mRNA expression of integrin receptors on monocytes/macrophages as counterparts to the endothelial adhesion molecules. Likewise, the mRNA levels of integrin α4β1 (very late antigen-4, *Vla4*), integrin αM (*Itgam*) and E-selectin ligand-1 (*Esl-1*) were slightly and transiently increased in the monocyte/macrophage cell line J774A.1 by MALP-2 (Figure S3). Consequently, the pretreatment of a monolayer of MyEnd cells with MALP-2 almost doubled the number of adherent J774A.1 cells (Figure 6c).

## 4. Discussion

Atherosclerosis, as a chronic inflammatory arterial disease, contributes to the major mortality of cardiovascular diseases worldwide. On the one hand, this is due to acute events such as myocardial infarction and strokes [23], but on the other, this is due to progressive lumen stenosis, which is the main trigger for adaptive arteriogenesis [4,5,6,18].

In this regard, growing collaterals represent a naturally occurring adaptive bypass system to avoid tissue ischemia. Well-developed collaterals, despite significant stenosis or even the occlusion of major coronary or peripheral arteries, could be the reason why some patients stay asymptomatic over a long period of time [4]. However, collateral growth is usually not sufficient to protect patients against ischemia for all their lives and thus therapies supporting this process are desirable. The model used in this study was a model of hind limb ischemia in mice. Critical limb ischemia represents the most severe form of PAD in patients [24,25]. The highly deadly disease is characterized by pain during walking and even at rest, as well as non-healing ulcers in the lower extremities. If the extent of the femoral artery occlusion due to advanced atherosclerosis becomes too large for percutaneous or surgical interventions, limb amputation remains the only treatment option. Catheter-based angiographic interventions or surgical bypasses are basically emergency procedures for the revascularization of the main artery in order to restore limb perfusion. Similar to these interventions, novel therapies such as cell-based or molecular therapies normally do not promote collateral growth [24,25]. Studies addressing therapeutic arteriogenesis are rare. Some of those investigated the potential of GM-CSF, identified in a rabbit model [20], with different outcomes in patients with coronary artery disease [26] or PAD [27]. Finally, the therapeutic improvement of collateral growth in cardiovascular patients hardly plays a role in clinical practice at present. In the current study, we used the lipopeptide and TLR2/6 ligand MALP-2 to investigate therapeutic arteriogenesis. Over the past few years, we had already demonstrated the high potential of MALP-2 to promote vascular regeneration, such as angiogenesis [14] and endothelial regeneration after vascular wounding [15]. We now identified the possible application of MALP-2 to promote arteriogenesis and uncovered the potential underlying mechanisms. We found that MALP-2 functionally improved perfusion recovery in the hind limb by enhanced collateral growth. The increase in the collateral lumen diameter was driven by augmented pericollateral macrophage accumulation and enhanced endothelial cell proliferation. MALP-2-enhanced the NO release of endothelial cells and improved NO-dependent vasorelaxation as well as endothelial adhesion molecule expression and subsequent monocytic cell adhesion. We had already reported enhanced secretion of GM-CSF from endothelial cells of various origin following MALP-2 stimulation [14,15]. Since the beneficial effect of GM-CSF on collateral growth has already been proven in animal experiments [20] and clinical studies [27], it is conceivable that the observed beneficial effect of MALP-2 on collateral growth is dependent on growth factors such as GM-CSF as well. Of note, we did not see any beneficial effects of MALP-2 application in two commonly used wild-type mouse strains—neither in C57BL/6 mice nor in BALB/c mice, which have known differences in cardiovascular regeneration [28]. As we saw the functional and morphological changes upon MALP-2 treatment that were summarized above exclusively in Apoe-deficient mice on a HFD and not in wild-type mice, we concluded that hypercholesteremic conditions are required for the beneficial MALP-2 effects on arteriogenesis. This conclusion was supported by the observation—to our knowledge, for the first time—that the collaterals were already positive for Oil Red O in this model. The staining demonstrated lipid deposition in the vascular wall of the collaterals, indicating vascular dysfunction. Ultimately, the mouse model used—with compromised vascular function and advanced atherosclerotic plaque load in larger arteries— approximately reflects the situation of cardiovascular patients.

In order to optimize the application route of MALP-2, we tested different variants. Initially, our intention was to choose an application route to bring MALP-2 as close as possible to the pre-existing collaterals after ligation. Therefore, we injected MALP-2 divided into small quantities into the Musculus adductor near to the collaterals. However, at the sites of injection, the tissue was affected in such a manner that subsequent histological analyses were not possible anymore. In addition, we tried to inject directly into the femoral artery proximal to the ligation. This application route proved difficult due to the small dimensions of the vessel. Since we observed increased mortality after the operation, we refrained from using this method. In the end, we chose the widely used intravenous application route (tail vein) for the MALP-2 injection, knowing that the lipophilic substance would be partially absorbed by the endothelium and that only small amounts would enter the target area of the collaterals. Although our approach was successful, there is still room to improve application strategies to bring MALP-2 into close proximity to the collaterals, e.g., in a biodegradable intra-arterial matrigel deposit or similar.

The potential limitations of our study are the same as those that generally apply for experimental studies in mice. The ligation of the femoral artery induces the growth of pre-existing collateral arteries and is therefore widely accepted as a reliable model for arteriogenesis. However, the vascular dimensions and related hemodynamic forces are different to the situation of cardiovascular patients. To substantiate our findings for a potential therapeutic use in promoting collateral growth, experiments in higher animals are needed. In regard to therapeutic angiogenesis, this has been already done in a sheep model of tissue engineering [16]. Moreover, we used just one single dose of MALP-2 (1 µg/mouse) as this was proved to be effective in a previous in vivo study by our group [15]. Dose-response experiments would maybe reveal an even more effective dose. However, based on the data already published, our local animal authorities did not approve dose-finding experiments in this study.

Seemingly, TLR2/6 signaling is particularly suitable in promoting vascular regeneration and adaptation. This is not only documented by our studies [14,15,16]. Indeed, other TLR2 ligands, such as bacterial peptidoglycan [29] or the proteoglycan versican as an endogenous ligand [30], have been shown to induce angiogenic factors. Likewise, endogenous lipid oxidation productions are capable of promoting angiogenesis [31]. The common principle of our studies is a single bolus injection of MALP-2 to transiently increase inflammation, which could be considered an immunological mechanism to promote regeneration and adaptation. In contrast, long-term application of MALP-2 led to increased circulating inflammatory markers and increased atherosclerosis [32].

In summary, we identified a novel property of the lipopeptide and TLR2/6 ligand MALP-2 to restore blood flow recovery by enhanced collateral growth with possible implications for therapeutic arteriogenesis (Figure S4).

## Supplementary Materials

The following are available online at https://www.mdpi.com/2073-4409/9/4/997/s1, Figure S1: Characterization of MyEnd cells, Figure S2: Vascular lipid deposition in hypercholesterolemic Apoe-KO 7 days after FAL and MALP-2 or PBS (control) injection, Figure S3: MALP-2 up-regulates mRNA expression for integrin receptors in J774A.1 cells, Figure S4: Mode of action of MALP-2 in arteriogenesis.
