# Supplementary material for: The Lipopeptide MALP-2 Promotes Collateral Growth
Source: Cells. 2020 Apr 16;9(4):997. doi: 10.3390/cells9040997 (PMC7227808; doi:10.3390/cells9040997)
Supplement: Supplementary file 1 [file cells-09-00997-s001.pdf]

- Supplemental Material -

Methods

Reagents

Phalloidin-iFluor 647 reagent was purchased from Abcam (Cambridge, UK).

Flow cytometry

MyEnd cells were characterized for their endothelial properties by flow cytometry. Briefly, cells were stained for 30 min on ice with antibodies against the endothelial cell marker CD31 (2.5 µg/mL) labelled with FITC. Cells were acquired on FACS LSR II flow cytometer (BD Biosciences, San Jose, CA) and analyzed using Flowjo (Tree Star Inc., Ashland, OR).

Histology

Vascular lipid deposition was identified in the aortic root, the thoracoabdominal aorta, the femoral artery and the collaterals by Oil Red O (Sigma-Aldrich) staining. The formalin-fixed thoracoabdominal aorta was stained with 0.6% propylenglycol-dissolved Oil Red O (2 h at 37°C) and then washed with 85% propylenglycol to remove excess dye and counterstained with hematoxylin solution (Gill No.2, Merck KGaA, Darmstadt, Germany). The remaining fat was removed under a stereomicroscope (Stemi DV4, Carl Zeiss Microimaging, Jena, Germany). Subsequently, the aorta was opened longitudinally, pinned on a black silicone-covered dish and photographed under PBS immersion using a stand-equipped camera (EOS 600D, Canon, Tokyo, Japan). Within the aortic root, serial cryostat sections (8 µm, CM3050S, Leica) at the level of all 3 cusps were prepared and aortic lipid deposition was analyzed at three distinct locations (in ~200, 500 and 800 µm distance from the aortic sinus) by Oil Red O staining (2 h at 60°C). Following the same protocol, serial sections of the femoral artery and the collaterals were likewise stained with Oil Red O.

Morphometric data were obtained using a light microscope (DMI3000 B microscope, Leica Microsystems Wetzlar, Germany) or a stand equipped with camera (EOS 600D Canon, Krefeld,

Germany) and ImageJ software (National Institutes of Health, Bethesda, MD). The atherosclerotic plaque size was determined by calculating the percentage of the Oil Red O positive area of the thoracoabdominal aorta or the total aortic root cross sectional area, respectively.

Figure and legends

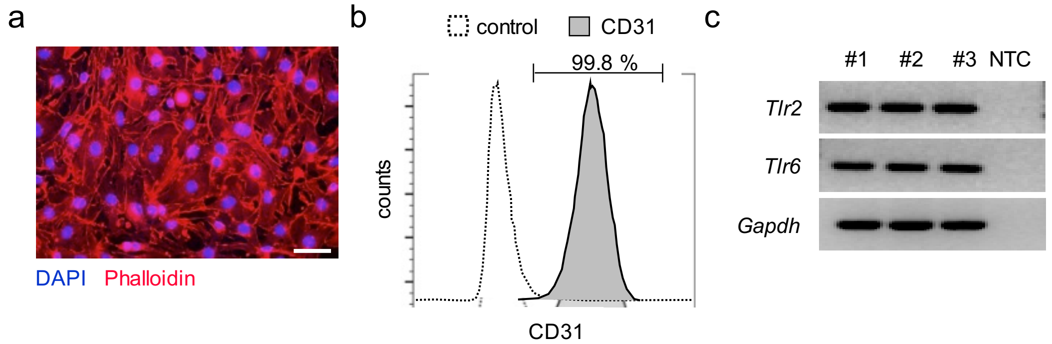

Figure S1 Characterization of MyEnd cells. (**a**) Fluorescence images depicting a monolayer of confluent grown cells. Cytoskeleton was stained with Phalloidin and cell nuclei with DAPI. Scale bar=5 µm (**b**) Cell surface expression of CD31 was verified by flow cytometry. (**c**) *Tlr2* and *Tlr6* mRNA expression was analyzed by real-time PCR and loaded onto agarose gels. *Gapdh* is shown as loading control. Representative pictures are shown. NTC=no template control.

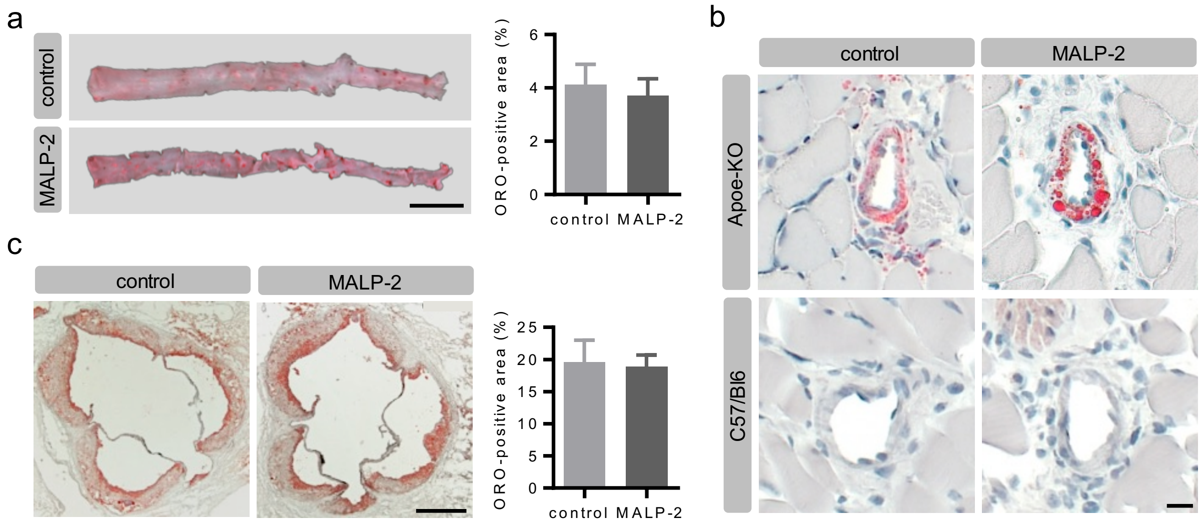

Figure S2 Vascular lipid deposition in hypercholesterolemic Apoe-KO 7 days after FAL and MALP-2 or PBS (control) injection. Oil Red O staining of (**a**) en face prepared thoracoabdominal aortas and sections of (**b**) the aortic root and (**c**) the collateral artery. Representative pictures and the corresponding quantitative analysis are shown. Oil Red O positive area is expressed as percentage of total surface area of the thoracoabdominal aorta or as percentage of total surface area of the aortic root. Scale bars 5 mm (**a**), 200 µm (**b**) and 25 µm (**c**), n=7.

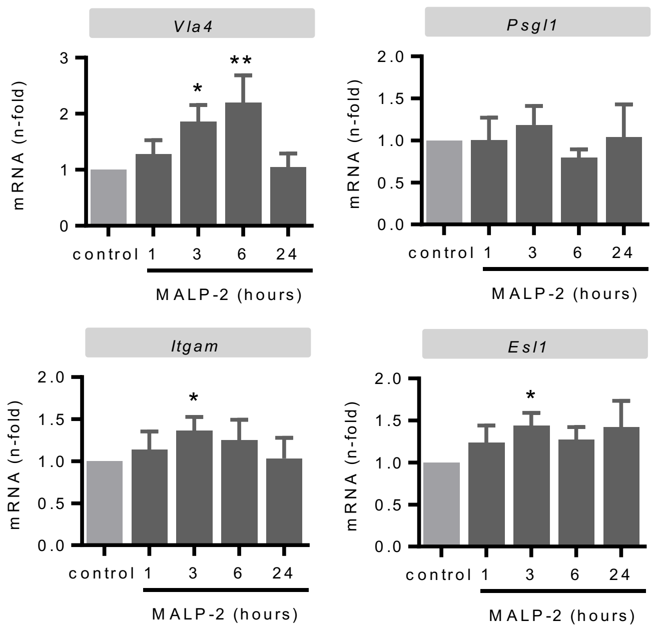

Figure S3 MALP-2 up-regulates mRNA expression for integrin receptors in J774A.1 cells. J774A.1 cells were stimulated with MALP-2 (1 µg/mL) and VLA4, PSGL1, ITGAM and ESL-1 mRNA levels were analyzed after the indicated time points by real-time PCR. *P<0.05, **P<0.01 vs. control, n=6-8.

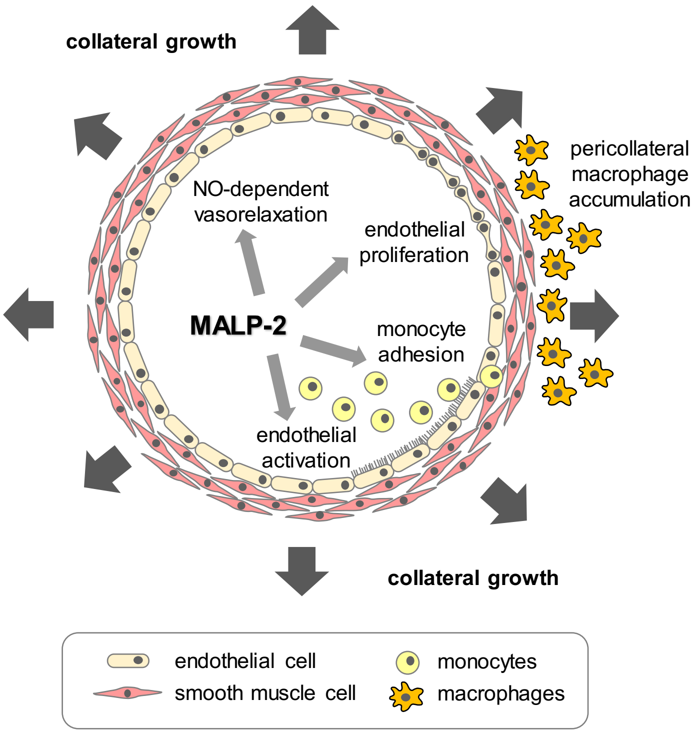

Figure S4 Mode of action of MALP-2 in arteriogenesis.
